# Protein disulfide isomerase in cardiovascular disease

**DOI:** 10.1038/s12276-020-0401-5

**Published:** 2020-03-18

**Authors:** Bei Xiong, Vishwanath Jha, Jeong-Ki Min, Jaehyung Cho

**Affiliations:** 1 Department of Pharmacology, University of Illinois-Chicago College of Medicine, Chicago, IL 60612 USA; 2 Department of Hematology, Zhongnan Hospital of Wuhan University, 430071 Wuhan, Hubei P.R. China; 3 Biotherapeutics Translational Research Center, Korea Research Institute of Bioscience and Biotechnology (KRIBB), Daejeon, Republic of Korea; 4 Department of Biomolecular Science, KRIBB School of Bioscience, Korea University of Science and Technology, Daejeon, Republic of Korea

**Keywords:** Mechanisms of disease, Translational research

## Abstract

Protein disulfide isomerase (PDI) participates in the pathogenesis of numerous diseases. Increasing evidence indicates that intravascular cell-derived PDI plays an important role in the initiation and progression of cardiovascular diseases, including thrombosis and vascular inflammation. Recent studies with PDI conditional knockout mice have advanced our understanding of the function of cell-specific PDI in disease processes. Furthermore, the identification and development of novel small-molecule PDI inhibitors has led into a new era of PDI research that transitioned from the bench to bedside. In this review, we will discuss recent findings on the regulatory role of PDI in cardiovascular disease.

## Exploring enzymes’ influence on cardiovascular disease

Efforts to untangle the functions of a large family of enzymes could lead researchers to new therapies for diverse cardiovascular diseases. Members of the protein disulfide isomerase (PDI) family chemically modify other proteins in ways that can alter both their structure and biological activity. Jaehyung Cho of the University of Illinois at Chicago, USA and coworkers have reviewed numerous studies linking PDI with cardiovascular diseases, including thrombosis, heart attack, vascular inflammation, and stroke. The authors also report progress in developing small-molecule PDI inhibitors that could yield the treatment for these conditions.

## Introduction

Cardiovascular diseases, including thrombosis, peripheral vasculitis, and stroke, are the leading cause of death in the U.S. and result in >30% of all deaths globally^1^. The increased adhesiveness of intravascular cells, such as platelets and leukocytes and the increased activity of coagulation factors, play central roles in the underlying pathology. Alterations in disulfide bonds in plasma proteins and cell surface molecules induce conformational changes and regulate their functions^2^. Because of their critical role in modifying thiol-disulfide bonds, thiol isomerases are involved in a broad range of cardiovascular diseases.

Protein disulfide isomerase (PDI or PDIA1) is a prototypic thiol isomerase that catalyzes the formation and cleavage of thiol-disulfide bonds during protein folding in the endoplasmic reticulum (ER)^3^. Despite the presence of 21 PDI family member thiol isomerases^3^, PDI (*P4HB*) knockout (KO) mice are embryonic lethal (our unpublished work), although the detailed mechanism remains to be elucidated. ERp57 (PDIA3), a PDI family thiol isomerase that has the same domain structure as and 34% sequence identity with PDI, cannot substitute for PDI^4^. The non-compensatory function of PDI is also found in yeast, which have four PDI-related genes^5,6^. These findings indicate that PDI is indispensable for the survival of organisms and that each oxidoreductase may act distinctly. PDI contains one TrpCysGlyHisCysLys active site in each of the two catalytic domains, which are essential for its oxidoreductase activity^7^. PDI also functions as a chaperone that prevents the formation of misfolded protein aggregates^8^. Although it has an ER retention sequence, PDI is released from a variety of cells, directly binds to cell surface molecules, and modulates their functions^9–12^. In addition to enriched expression in the pancreas and liver, PDI is also widely expressed in other tissues^13^. Intriguingly, the expression level of PDI is altered in various cancers and neurodegenerative disorders^14,15^. Studies using tissue-specific PDI conditional KO (CKO) mice have demonstrated that intravascular cell-derived PDI contributes to the pathology of thrombosis, inflammation, and thromboinflammation^11,12,16^. Since PDI family member thiol isomerases have recently been reviewed elsewhere^17,18^, this review will focus on the pathophysiological role of PDI in cardiovascular disease.

## Structure of PDI

**Fig. 1:**
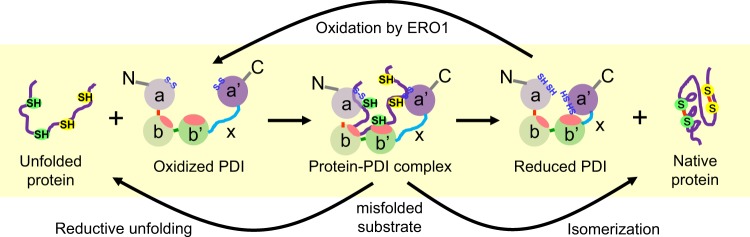
Protein folding by PDI in the ER. Oxidizing equivalents are transferred from the active site disulfide bonds (Cys53-Cys56 and Cys397-Cys400) of oxidized PDI to the reduced substrate. In turn, PDI is reduced and reoxidized by ERO1 in the ER environment. Misfolded substrates are reduced and reoxidized or directly isomerized. The x-linker region (X) is also indicated. The highly acidic region (c) is not shown here. N and C represent the N- and C-terminus, respectively.

PDI has two catalytically active a and a′ domains that are separated by two catalytically inactive b and b′ domains, the latter of which contains a hydrophobic substrate-binding region (Fig. 1)^7^. In the ER, PDI transfers oxidizing equivalents to an unfolded substrate, facilitating protein folding. Misfolded protein substrates are reduced and re-oxidized or directly isomerized. A flexible 19 amino acid peptide (x-linker) is located between the b’ and a’ domains. The C-terminal region of PDI contains 18 acidic residues, which play a role in the stabilization and maintenance of the functional conformation of PDI and in the prevention of self-aggregation^19^. There is an ER retention sequence (LysAspGluLeu) at the C-terminus. The crystal structure of human PDI reveals that the TrpCysGlyHisCysLys active site in the a and a′ domains faces each other at the entrance of the substrate-binding pocket^20^. While the distance between the sulfur atoms of Cys53 in the a domain and Cys397 in the a′ domain is 27.6 Å in reduced PDI, the distance in oxidized PDI increases to 40.3 Å^20^. Moreover, the flexible x-linker region caps and uncaps a hydrophobic site on the b′ domain which controls substrate binding^21,22^. Binding of bepristats (small-molecule PDI inhibitors) to the b′ domain of PDI displaces the x-linker and paradoxically increases the reductase activity at the a and a′ domains^23^. Given the different redox environments inside (highly oxidizing) and outside (reducing) of the ER, the enhancement of PDI reductase activity by drug or substrate binding to the substrate binding pocket may occur in non-ER locations. Oxidative stress induced by reactive oxygen species (ROS) influences the local redox environment and plays a critical role in the development of a wide range of cardiovascular diseases^24^. The altered redox environment during the disease condition is likely to affect the oxidoreductase activity of PDI. Taken together, these findings suggest that a disease-related redox switch induces a conformational change in extracellular PDI and regulates substrate binding.

## Source and function of extracellular PDI

Although primarily localized in the ER, a small portion of mammalian PDI is found in the nucleus, cytosol, cell surface, and extracellular space^25^. It was recently reported that the loss of luminal ER calcium results in an exodus of ER-resident proteins, including PDI, and alters the composition of the ER luminal proteome and secretome^26^. Furthermore, cell surface trafficking of PDI depends on the LysAspGluLeu receptor 1, which is detectable on the cell surface but is different from agonist-induced secretion into the extracellular space^27^. In addition to the ER, PDI is localized in and released from secretory granules or vesicles. Immunogold electron microscopy with platelets demonstrated the localization of PDI in the T-granule, a novel electron-dense tubular system-related compartment^28^. Proteomic analysis showed that PDI is found in secondary (specific) and tertiary (gelatinase) granules in neutrophils^29^. In contrast, another study revealed that neutrophil PDI is localized predominantly in the primary (azurophilic) granule and cytosolic fractions^30^. Using high-resolution immunofluorescence microscopy, Crescente et al. reported that PDI is localized in a compartment that is different from known secretory granules and translocates to the surface of activated platelets in a manner that is dependent on actin polymerization but not Munc13-4-mediated membrane fusion^31^. A study using platelets that were isolated from mice lacking the *HPS6* gene and patients with Hermansky-Pudlak syndrome revealed that ADP released from dense granules is required for PDI secretion from T-granules^32^. In endothelial cells, PDI is stored in a vesicle that is distinct from Weibel-Palade bodies^33^, and disruption of actin or microtubule polymerization or arterial shear stress enhances PDI secretion^34^. Intriguingly, vascular smooth muscle cell PDI is detected as a cell surface-bound but not soluble form after secretion by Golgi-independent routes^35^. Altogether, these findings indicate that each cellular PDI might be secreted through a distinct mechanism. However, it remains unclear whether extracellular PDI originates from PDI that escapes from the ER or is released from secretory granules or vesicles under disease conditions.

Cell-released PDI is found on the plasma membrane and regulates the function of cell surface molecules. Studies using blocking antibodies and cell-impermeable thiol-reacting agents suggest the importance of cell surface-bound PDI the ligand-binding function of cell surface molecules such as integrins^10–12,36–38^. Furthermore, the use of small-molecule PDI inhibitors helped to examine how PDI interacts with its binding partners and modulates cellular functions^23,39,40^ and to demonstrate that targeting PDI could be a novel therapeutic strategy for treating thrombotic disease^41^. However, it should be noted that many PDI inhibitors and even blocking antibodies (e.g., clone RL90), which have been used in numerous studies, have off-target effects or cross-reactivity with other thiol isomerases^10,12,16,42^. In addition, there is a possibility that small-molecule inhibitors enter cells and perturb the critical function of intracellular PDI. Genetic approaches, therefore, have been employed to determine the role of specific cell-derived PDI. Studies using PDI CKO mice and recombinant wild-type and oxidoreductase activity-null mutant PDI have demonstrated that platelet- and neutrophil-released PDI directly binds to αIIbβ3 and αMβ2 integrins, respectively and that the oxidoreductase activity of cell surface-localized PDI plays a crucial role in promoting the ligand-binding activity of integrins during cell activation^10–12^. Given the intrinsic function of PDI in the ER, it has been hypothesized that extracellular PDI facilitates the formation or cleavage of disulfide bonds in cell surface molecules, induces conformational changes or clustering, and alters their function in cardiovascular disease.

## Cell surface molecules targeted by extracellular PDI

The functions of plasma proteins and cell surface molecules are regulated by oxidation or reduction of allosteric disulfide bonds. These disulfide bonds are identified by secondary structural motifs, surface exposure, and three configurations (−RHStaple, −LHHook or ±RHHook)^2^. A recent review from Chui and Hogg discusses the unique features of allosteric disulfide bonds^43^. Extracellular PDI and other oxidoreductases are major modifiers of disulfide bonds. Plasma proteins and cell surface molecules whose functions are regulated by extracellular PDI include thrombospondin 1^44^, vitronectin^45^, integrins^10,11,46^, and glycoprotein Ibα (GPIbα) of the GPIb-IX-V complex^16^. In particular, the role of extracellular PDI in the ligand-binding activity of α2β1, αIIbβ3, and αMβ2 integrins has been demonstrated^10,11,38^. However, the detailed molecular mechanism by which PDI modulates integrin function is still unclear. Mass spectrometric analysis revealed that only the Cys177-Cys184 disulfide bond in the β3 subunit of αIIbβ3 integrin is cleaved by ERp5 but not PDI^47^, raising the possibility that thiol isomerases modify distinct disulfide bonds within the same molecule. In support, treatment with a blocking anti-ERp57 antibody further impaired αIIbβ3 integrin activation and aggregation of PDI null platelets, indicating the distinct role of ERp57 and PDI^10^. Similar mass spectrometric techniques could be used to reveal the PDI-targeted disulfide bonds in other integrins. A study using trapping PDI showed that PDI binds to various platelet-derived molecules, including glutaredoxin-1, thioredoxin, fibrinogen, heparanase, ERp57, kallikrein-14, serpin B6, and tetranectin^48^. Such an approach would help identify the Cys residues that are responsible for PDI regulation of target molecules.

Bioinformatic analysis using a database on disulfide bonds (http://149.171.101.136/python/disulfideanalysis/index.html)^49^ showed that many platelet receptors involved in platelet activation and adhesion contain one or more putative allosteric disulfide bonds which have not been reported^16^. These include GPIbα/β, toll-like receptors, and CD40. Consistently, we found that platelet PDI directly binds to GPIbα and cleaves two allosteric Cys4-Cys17 (-RHStaple) and Cys209-Cys248 (-LHHook) disulfide bonds, inducing conformational changes and enhancing the ligand-binding function^16^. Furthermore, our in vivo studies revealed that PDI-regulated GPIbα function is crucial for vascular occlusion and tissue damage under thromboinflammatory conditions, such as vasculitis, stroke, and sickle cell disease^16^. This study has advanced our understanding of the molecular mechanism by which platelet-released PDI promotes GPIbα function and participates in the pathogenesis of thromboinflammation.

## Regulators of extracellular PDI activity

Although extracellular PDI contributes to the pathogenesis of cardiovascular disease, it is not known how extracellular PDI activity is regulated under disease conditions. As key post-translational modifications that affect numerous signaling pathways, S-nitrosylation and S-glutathionylation of proteins occur during nitrosative or oxidative stress^50,51^. Like the two active TrpCysGlyHisCysLys sites of PDI, Cys residues that are adjacent to a basic environment (i.e., Lys, Arg, or His) have low pKa values and are targeted for covalent modification. In the ER, S-nitrosylation, S-glutathionylation, and S-mercuration occur on the active sites of PDI, inhibiting its enzymatic activity and promoting the unfolded protein response and ER stress^15,52,53^. Uehara et al. reported that PDI is S-nitrosylated in brain tissues from patients with Parkinson’s and Alzheimer’s diseases and that S-nitrosylation impairs the protective effect of PDI on neurotoxicity that is induced by protein misfolding in neurodegenerative disorders^15^. Furthermore, there is evidence that S-nitrosylation occurs in extracellular PDI. Human erythroleukemia cell surface-bound PDI is S-nitrosylated and then transfers nitric oxide (NO) into the cell^54^. Conversely, PDI exports NO from red blood cells^55^. S-nitrosylated PDI transverses the plasma membrane of red blood cells, strongly attaches to the cell surface under normoxia, and binds to endothelial cells after entering the tissues, resulting in NO release and vasodilation^55^. A recent study revealed that NO-mediated S-nitrosylation of platelet and endothelial cell PDI reduces its reductase activity and inhibits the prothrombotic function of these cells^56^. Taken together, these results suggest that S-nitrosylation allows PDI to transfer NO in and out of cells and negatively regulates the oxidoreductase activity of extracellular PDI.

S-glutathionylation also acts as a biological switch due to its reversibility and modulates oxidative signaling events^51^. As a critical antioxidant, reduced glutathione is present in millimolar concentrations (0.1–10 mM) in cells and ~0.85 mM in the blood of healthy people^57,58^. While the blood level of reduced glutathione is not different between healthy people and patients with cardiovascular disease, the level of free plasma Cys is significantly increased in patients^59^. Mass spectrometric analysis showed that nitrosative stress induces S-glutathionylation but not S-nitrosylation of both active site Cys residues in PDI^60^. In the ER, nitrosative stress-induced S-glutathionylation of PDI induces activation of the unfolded protein response. Although the ratio of reduced to oxidized glutathione is a major contributor to cellular redox potential and homeostasis and is changed under disease conditions, it is not known whether PDI is S-glutathionylated in the extracellular milieu.

ER oxidoreductin 1 (ERO1) oxidizes PDI via disulfide bond exchange in the ER, enabling PDI to oxidize or isomerize disulfide bonds in substrate proteins (Fig. 1)^61^. Of the two isoforms found in mammalian cells, ERO1α is ubiquitously expressed^62^, whereas ERO1β is predominantly found in the stomach and pancreas^63,64^. Unlike PDI KO mice, ERO1α/β KO mice are viable but exhibit delayed protein folding^65^, indicating the role of ERO1 in disulfide bond oxidation during protein folding, as well as the presence of an ERO1-independent mechanism of PDI oxidation (e.g., peroxiredoxin 4-mediated PDI oxidation)^66,67^. An in vitro study revealed that ERO1α is associated with PDI and αIIbβ3 in platelets and that treatment with polyclonal anti-ERO1α antibodies inhibits agonist-induced αIIbβ3 activation and platelet aggregation^68^. Although these findings suggest that platelet-released ERO1α regulates platelet function, it is unknown whether ERO1α plays a role in thrombosis in vivo.

The oxidoreductase activity of PDI is controlled by the local redox environment. ROS produced by NADPH oxidases (NOXs) are major contributors to oxidative stress and alter the redox environment. A study showed that PDI interacts with p47^phox^, a cytosolic subunit of NOX1 and NOX2, through a disulfide bond in PMA-activated neutrophils, indicating a role for PDI in ROS generation^30^. However, deletion of neutrophil PDI did not affect O_2_^•–^/H_2_O_2_ generation upon agonist stimulation (our unpublished work). The PDI-p47^phox^ interaction mediated by intermolecular disulfide bonds also occurs in vascular smooth muscle cells (VSMCs) and is required for NOX1 activation^69^. Thus, further studies are required to investigate the relationship between PDI and NOX-produced ROS.

## Small-molecule inhibitors of PDI

A recent advance in PDI research is the identification of novel small-molecule inhibitors. A previous study identified quercetin-3-rutinoside (rutin, a flavonoid antioxidant) as a reversible PDI inhibitor with a Kd value of 2.8 μM^70^. Rutin at 45–60 μM inhibited AlaTyrProGlyLysPhe (protease-activated receptor 4-activating peptide)-induced platelet aggregation by 60–90% of the vehicle control^70^. Intravital microscopic studies showed that intravenous injection of rutin (0.5 mg/kg) abrogates platelet thrombus formation and fibrin generation at the site of laser-induced arteriolar injury and increases the time to occlusion in a FeCl_3_-induced cremaster arteriolar thrombosis model^70^. Since rutin binds to the PDI b’ domain, intravenous injection of the b′ domain reverses its antithrombotic effect in mice^40^. However, another study showed that 30 μM rutin equivalently inhibits both PDI and ERp57 activities in a cell-free system^12^. Furthermore, studies using platelets isolated from megakaryocyte-specific PDI CKO mice demonstrated that rutin at 50 μM exhibits a significant inhibitory effect on platelet aggregation induced by thrombin or von Willebrand factor^10,16^. These results indicate that rutin has off-target effects at concentrations that inhibit PDI activity. Currently, isoquercetin, a flavonoid quercetin, is being evaluated as an antithrombotic agent in clinical studies^41^.

High throughput screening (HTS) of 348,505 compounds identified ML359 and bepristats as potent PDI inhibitors^23,39^. Despite the strong IC_50_ value of 0.25 μM in a cell-free system, 30 μM ML359 minimally inhibited thrombin-induced platelet aggregation (25%), indicating the poor biochemical property and efficiency of the compound^71^. The site at which ML359 binds to PDI is unknown. Bepristat 2a binds to the b′ domain of PDI, and its binding to the substrate-binding region enhances the catalytic activity of the two active sites by displacing the x-linker^23^. This finding provides insight into the molecular mechanism by which substrate binding alters PDI catalytic activity.

**Fig. 2:**
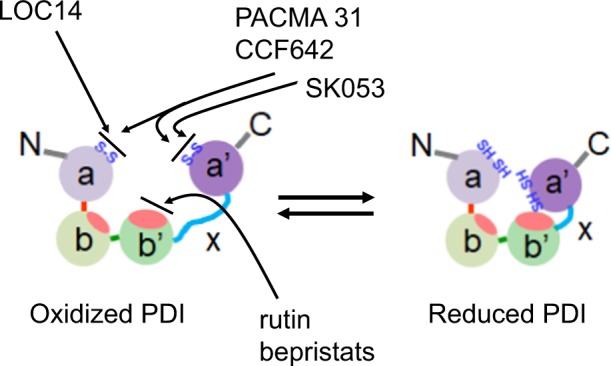
PDI activity in the extracellular region. As the redox environment outside the cell is reducing compared to the highly oxidizing ER environment, PDI is likely to function as a reductase in the extracellular milieu. The binding sites of some PDI inhibitors to oxidized PDI are described.

PDI inhibitors have also been identified as neuroprotective and anti-cancer agents. Using HTS of 10,000 compounds, Kaplan et al. identified lead optimized compound 14 (LOC14) as a reversible and potent PDI inhibitor (Kd = 62 nM) and confirmed the neuroprotective effect in corticostriatal brain slice cultures^72^. LOC14 binds to the active site of the a domain of PDI, is stable in mouse liver microsomes and blood plasma, and exhibits low plasma-protein binding. The compound penetrates the blood-brain barrier^72^, and chronic administration of LOC14 provides neuroprotective effects and suppresses ER stress in a mouse model of Huntington’s disease^73^. SK053, PACMA 31, and CCF642 have been identified as PDI inhibitors for treating acute myeloid leukemia, ovarian cancer, and multiple myeloma, respectively^74–76^. SK053 binds to the C-terminal active site of PDI and inhibits the enzymatic activity with an IC_50_ value of 10 μM, exhibiting leukemic effects^74^. PACMA 31 is an irreversible PDI inhibitor with an IC_50_ value of 10 μM^75^. It covalently binds to the Cys397 and Cys400 residues of the a′ domain. PACMA 31 exhibits cytotoxicity to ovarian cancer cell lines in vitro and human ovarian cancer cell growth in a mouse xenograft model after intraperitoneal or oral administration, without causing toxicity to normal tissues^75^. CCF642 was identified as a PDI inhibitor with a submicromolar IC_50_ value using a mechanistically unbiased algorithm on a library of 30,335 small molecules^76^. Drug-protein docking modeling suggests that covalent binding occurs between the Lys401 (and possibly Lys57) residue of PDI and the carbonyl group of CCF642. The compound has potent effects against multiple myeloma activity that are comparable to those of bortezomib, a first-line multiple myeloma therapeutic. Other studies also identified novel PDI inhibitors (e.g., juniferdin^77^, origamicin^78^, 16F16^79^, securinine^80^, P1^81^, and 35G8^82^) to treat HIV-1 infection, neurodegenerative diseases, or glioblastoma. Figure 2 illustrates the binding sites of some PDI inhibitors. Although the specificity, cell-permeability, and in vivo efficacy of these drugs should be further investigated, it would be of interest to test whether they have antithrombotic efficacy.

## Role of PDI in the pathology of cardiovascular disease

PDI participates in the initiation and progression of numerous cardiovascular diseases. Although many studies have been conducted with pharmacological inhibitors, their non-specific or off-target effects have made the PDI CKO mouse an essential tool to study the precise molecular mechanisms of PDI. Here, we will discuss the recent findings related to the contribution of extracellular PDI to thrombosis and vascular inflammation. In addition, we will summarize the role of intracellular PDI in the pathogenesis of myocardial infarction, stroke, and atherosclerosis.

## Thrombosis

Since previous work in the 1990s identified that PDI plays a role in platelet functions^83–85^, many efforts have been made to elucidate the molecular mechanism by which platelet PDI contributes to arterial thrombosis. The first evidence of the in vivo function of PDI was reported using a blocking anti-PDI antibody (RL90), which suggested that inhibition of extracellular PDI activity reduces platelet thrombus formation and fibrin generation following laser-induced cremaster arteriolar injury in mice^86^. However, the antithrombotic effect was accompanied by prolonged tail bleeding times and increased blood loss at the site of tail amputation. Although it is unclear whether treatment of mice with the anti-PDI antibody inhibited the activity of other thiol isomerases such as ERp57 in vivo, these results raised concerns that specific PDI inhibitors may impair hemostatic functions. Studies with megakaryocyte-specific PDI CKO mice demonstrated that the loss of platelet PDI reduces full activation of αIIbβ3 integrin after agonist stimulation and attenuates platelet thrombus formation in vivo^10,12^. Due to the discrepancy between these two studies, however, it remains to be further determined whether platelet PDI is involved in granule secretion and hemostasis. It was recently reported that PDI-bearing endothelial cell microparticles isolated from diabetic mice activate platelet αIIbβ3 integrin^87^, suggesting that endothelial cell-derived PDI plays a role in platelet activation under diabetic conditions. However, it should be noted that platelet thrombus formation was significantly reduced even in the presence of PDI that was derived from other intravascular cells in megakaryocyte-specific PDI CKO mice^10,12^. This may result from the spatial separation between other cell-released PDI and αIIbβ3 integrin on aggregating platelets or rapid washout of the released PDI by blood flow. Although endothelial cell-released PDI is likely responsible for fibrin generation at the site of laser-induced arteriolar injury^33^, it should be examined whether PDI derived from other intravascular cells plays a role in arterial thrombosis. Another study showed that extracellular PDI cleaves disulfide bonds in plasma vitronectin, enabling vitronectin to interact with β3 integrin and promote arterial thrombosis^45^.

Unlike arterial thrombosis, which is mainly composed of platelets, venous thrombosis is enriched in fibrin and erythrocytes. PDI and tissue factor are upregulated in leukocytes and endothelial cells in an inferior vena cava ligation model in rats^88^. A recent study using a mouse model of inferior vena cava partial stenosis showed that blocking PDI with PACMA 31 inhibits both platelet deposition and fibrin formation in a manner that depends on tissue factor on myeloid cells^89^. Complement factors are crucial for venous thrombosis; C3 contributes to platelet and tissue factor procoagulant activation, and C5 is crucial for exposure of leukocyte procoagulant phosphatidylserine^89^. Muller-Calleja et al. demonstrated that tissue factor activation is reduced by 16F16 (a PDI inhibitor) or 10H10 (an anti-tissue factor antibody that blocks PDI-tissue factor binding) and requires C3 but not C5^90^. Although this report is consistent with the hypothesis that PDI decrypts tissue factor by oxidizing the Cys186-Cys209 disulfide bond or through its chaperone activity, several studies undermined the hypothesis^91–93^.

## Vascular inflammation

As the most abundant leukocyte, neutrophils are recruited to inflamed tissues through rolling, adhesion, crawling, and transmigration, killing bacteria or inducing tissue injury^94,95^. Bennett et al. reported that neutrophil PDI may influence l-selectin shedding by regulating the activity of tumor necrosis factor-α-converting enzyme^96^, suggesting a role of PDI in neutrophil rolling in inflammation. However, neutrophils isolated from myeloid-specific PDI CKO mice had no effect on l-selectin shedding^11^. Introducing pairs of Cys residues into the I domain of αM and αL integrin subunits affects the ligand-binding function of β2 integrins^97,98^. Neutrophil-released PDI directly binds to activated αMβ2 integrin and alters thiol exposure in the αM subunit, promoting ligand-binding activity and neutrophil recruitment during vascular inflammation^11^. However, it remains to be determined which Cys residues in the integrin are modified by neutrophil PDI.

Mounting evidence indicates that neutrophils adhere to inflamed endothelial cells to support platelet adhesion mainly through the interactions of neutrophil P-selectin glycoprotein ligand-1 and αMβ2 integrin with platelet P-selectin and GPIbα, respectively^99^. Heterotypic cell-cell interaction at the site of vascular injury induces the release of prothrombotic and proinflammatory molecules^100^, exacerbating inflammatory conditions. Our recent studies demonstrated that platelet-released PDI positively regulates the ligand-binding function of GPIbα by direct interaction and enhances GPIbα-mediated platelet adhesiveness and platelet–neutrophil interactions during sterile vascular inflammation^16^. Treatment of mice with a blocking anti-PDI antibody or anfibatide, a clinical-stage GPIbα antagonist, markedly inhibited platelet–neutrophil interactions and mitigated thromboinflammatory conditions. These results provide evidence that the PDI-GPIbα signaling axis could be a novel therapeutic target for the treatment of thromboinflammatory disease.

## Myocardial infarction

Acute myocardial infarction induces activation of the unfolded protein response after ER stress, leading to cardiomyocyte apoptosis and death^101^. A recent study using a left anterior descending artery ligation mouse model of acute myocardial infarction showed that PDI expression is enhanced in the infarcted area^102^. Severino et al. demonstrated that PDI is upregulated in autoptic infarcted hearts obtained from 18 patients and that PDI is upregulated in cardiomyocytes after hypoxic stress and protects the cells from apoptosis^103^. Adenovirus-mediated transfer of the *P4HB* gene to the mouse heart significantly reduced the infarct size and cardiomyocyte apoptosis in the peri-infarct region. These results suggest that pharmacological modulators of PDI expression might be useful in preventing and treating heart failure. Using bioptic myocardial tissue sections harvested from diabetic and nondiabetic patients and mice, the same group reported that diabetic conditions alter the redox state of ischemia-induced PDI, which may account for the lack of a protective effect of PDI in diabetic hearts^104^. Another study using a mouse cardiomyocyte cell line suggested that upregulated PDI increases superoxide dismutase 1 activity without affecting the protein expression, protecting myocardial tissue from superoxide-induced apoptosis^105^. PDI is also upregulated in myocardial capillary endothelial cells in the viable peri-infarct and infarct regions of mouse hearts after chronic hypoxia^106^. PDI knockdown in human umbilical vein endothelial cells significantly increases the number of apoptotic cells and reduces cell migration and adhesion and tubular formation in both normoxic and hypoxic conditions. These results suggest that PDI protects cardiomyocytes and endothelial cells from apoptosis under hypoxia.

## Ischemic stroke

Under hypoxic conditions, PDI is upregulated in glia in vitro and in the cerebral cortex after transient forebrain ischemia in rats and protects against hypoxia-induced cell death in a manner that is dependent on its oxidoreductase activity^107^. Consistently, studies using a rat model of ischemia/reperfusion-induced stroke suggested that upregulation of PDI exhibits the cytoprotective effect of p-hydroxybenzyl alcohol and tanshinone IIA in the brain^108,109^. In contrast, proteomic analysis revealed that lipid-lowering agents such as atorvastatin protect from the sequelae of brain ischemic stroke by inhibiting the overexpression of PDI^110^. Our recent study demonstrated that specific deletion of platelet PDI decreases the infarct volume by mitigating thromboinflammatory conditions and improves neurological deficits in a mouse model of middle cerebral artery occlusion/reperfusion-induced stroke. Since previous studies showed upregulation of PDI in the cerebral cortex and its cytoprotective role in ischemic stroke, these results suggest that each cellular PDI plays a distinct role in the pathology of stroke.

## Atherosclerosis

Oxidized low-density lipoproteins (oxLDLs) are a major contributor to atherogenesis, triggering cellular activation, proliferation, and inflammation^111^. Migration and proliferation of VSMCs play a crucial role in atherosclerosis^112^. PDI knockdown in VSMCs decreases platelet-derived growth factor-induced ROS and cell migration by suppressing the increase in expression of NOX1. In contrast, PDI overexpression increases spontaneous basal migration of VSMCs^113^. Biochemical studies suggested that PDI interacts with RhoGDI and alters platelet-derived growth factor-induced Rac1 and RhoA activities^113^. Mechanical stretch stress and advanced glycosylation end products trigger proliferation and apoptosis of VSMCs^114^. A recent study revealed that both stretch stress and advanced glycosylation end products synergistically upregulate PDI in VSMCs and that PDI upregulation increases cell proliferation and apoptosis, leading to diabetic vein graft atherosclerosis^115^. These results suggest that PDI may be a novel therapeutic target for the treatment of vascular remodeling and atherosclerosis. Treatment of human microvascular endothelial cells with oxLDL inhibits PDI reductase activity, and overexpression of wild-type but not oxidoreductase activity-null PDI in endothelial cells reduces oxLDL-induced ER stress and toxicity^116^. Furthermore, PDI modification by lipid peroxidation products occurs in endothelial cells and the macrophage-rich core of advanced atherosclerotic lesions, suggesting a possible loss of function of PDI in atherosclerosis.

## Conclusions

Each cellular PDI functions differently in the pathogenesis of numerous cardiovascular diseases. Studies using tissue-specific PDI CKO mice have provided mechanistic insights into the role of platelet- and neutrophil-derived PDI in disease conditions. Furthermore, bioinformatics and advanced mass spectrometric technology have had a tremendous impact on the field of PDI research by identifying PDI-modified allosteric disulfide bonds. Our recent studies using bioinformatics, mass spectrometry, biochemical and cell biological approaches, and animal disease models have laid the groundwork for studying the molecular mechanism by which other thiol isomerases regulate the function of target molecules under disease conditions. These findings also raise several questions to be addressed. Which allosteric disulfide bonds in cell surface molecules are targeted by extracellular PDI and other oxidoreductases? How do PDI and its family member thiol isomerases alter the function of the same molecule, such as platelet αIIbβ3 integrin, and do they modify the same or different disulfide bonds? As PDI has numerous intracellular and extracellular functions, is blocking a specific PDI signaling pathway (e.g., the PDI-GPIbα signaling axis) better than conventional inhibition of PDI? Although there is no known inherited disorder that represents PDI deficiency or mutation in humans, posttranslational modification of PDI (e.g., S-nitrosylation) is associated with neurodegenerative diseases. Therefore, understanding how extracellular PDI activity is controlled in cardiovascular disease would be of particular importance.
